# Detection of multiple quantitative trait loci and their pleiotropic effects in outbred pig populations

**DOI:** 10.1186/1297-9686-41-44

**Published:** 2009-10-06

**Authors:** Yoshitaka Nagamine, Ricardo Pong-Wong, Peter M Visscher, Chris S Haley

**Affiliations:** 1 National Institute of Livestock and Grassland Science, Tsukuba, 305-0901, Japan; 2 The Roslin Institute (The University of Edinburgh), Midlothian, EH25 9PS, UK; 3 Queensland Institute of Medical Research, Brisbane, QLD, 4029, Australia; 4 Human Genetics Unit, Medical Research Centre, Edinburgh, EH4 2XU, UK

## Abstract

**Background:**

Simultaneous detection of multiple QTLs (quantitative trait loci) may allow more accurate estimation of genetic effects. We have analyzed outbred commercial pig populations with different single and multiple models to clarify their genetic properties and in addition, we have investigated pleiotropy among growth and obesity traits based on allelic correlation within a gamete.

**Methods:**

Three closed populations, (A) 427 individuals from a Yorkshire and Large White synthetic breed, (B) 547 Large White individuals and (C) 531 Large White individuals, were analyzed using a variance component method with one-QTL and two-QTL models. Six markers on chromosome 4 and five to seven markers on chromosome 7 were used.

**Results:**

Population A displayed a high test statistic for the fat trait when applying the two-QTL model with two positions on two chromosomes. The estimated heritabilities for polygenic effects and for the first and second QTL were 19%, 17% and 21%, respectively. The high correlation of the estimated allelic effect on the same gamete and QTL test statistics suggested that the two separate QTL which were detected on different chromosomes both have pleiotropic effects on the two fat traits. Analysis of population B using the one-QTL model for three fat traits found a similar peak position on chromosome 7. Allelic effects of three fat traits from the same gamete were highly correlated suggesting the presence of a pleiotropic QTL. In population C, three growth traits also displayed similar peak positions on chromosome 7 and allelic effects from the same gamete were correlated.

**Conclusion:**

Detection of the second QTL in a model reduced the polygenic heritability and should improve accuracy of estimated heritabilities for both QTLs.

## Background

QTLs (quantitative trait loci) in pigs are generally detected with the F_2 _design [1,2] because the power of detection using the line-cross methodology is greater than that using within-population data [3,4]. However, following the QTL reports of Evans *et al*. [5] and Nagamine *et al*. [6] in European commercial pig breeds, several authors have identified QTLs in outbred pig populations [7-9]. To date, several different analysis methods have been applied. The groups of Evans [5] and Nagamine [6] have used the half-sib regression method [10,11], those of de Koning *et al*. [12] and Nagamine *et al*. [13,14] the variance component analysis [15,16] and recently, Varona *et al*. [8] have performed a Bayesian analysis [17,18]. One of the advantages of variance component and Bayesian analyses is that these methods explicitly consider not only targeted QTLs but also residual polygenic effects in complicated pedigrees.

It is natural to assume that any detected QTL is one of the loci contributing to the polygenic component affecting the trait. Nagamine and colleagues [14] have reported the heritabilities of QTLs and residual polygenic effects using the variance component analysis with a one-QTL model, where they indicated that the detected QTL had relatively large effects. However, the heritability of residual polygenic effects on several traits was greater than that from the detected QTL. Simultaneous detection of QTLs using the multiple QTL model may reduce the residual polygenic variance and allow a more accurate estimation of QTLs and polygenic gene effects.

It is also important to consider the potential multiple effects of a QTL. For instance, pleiotropy is a phenomenon whereby a single gene affects two or more characteristics [19]. High phenotypic and polygenic correlations are often reported among growth or obesity traits *e.g*. [20,21]. It is reasonable to assume that a QTL may often act on related traits. Nevertheless, pleiotropic effects of QTLs acting on multiple traits have seldom been investigated in domestic animals. A few reports on pigs have been documented [22,23] using line crosses (*e.g*. F_2 _cross). If two linked QTLs are very close, it is difficult to judge whether two separate but linked loci are present or, alternatively, whether there is one QTL acting on two traits [24,25]. In a QTL study based on a cross between two lines, the cross generates strong linkage disequilibrium (LD) between linked loci, thus making it very difficult to distinguish linkage from pleiotropy. However, this is not generally the case in an outbred population where LD is usually limited and therefore, a strong correlation between allelic effects of the same parent gamete on two traits can suggest the evidence for pleiotropic effects.

Previously, we have detected significant QTLs for growth and obesity traits using least squares [6] and variance component analyses [13,14] on two chromosomes, 4 and 7, in modern commercial pig populations. In this report, we re-analyze these outbred pig populations using two-QTL models to clarify the genetic relationship between polygenes and genes at two QTLs. In addition, we investigate pleiotropy among growth and obesity traits based on allelic correlation on a gamete.

## Methods

### Data

Animals from three populations were analysed: (A) 427 individuals from a Yorkshire and Large White synthetic breed, (B) 547 Large White individuals, and (C) 531 Large White pigs individuals (Table 1). The populations were structured as half-sib families and the numbers of sires, dams and progeny across the populations ranged from 10 to 11, 91 to 146, and 326 to 391, respectively. Groups A, B and C had been maintained as closed populations for at least 15, 10 and 16 generations, respectively prior to sampling. Two fat traits (back fat P1 and P2) were analysed in population A and three fat traits (back fat P1 and P3 and loin fat L) in population B. Three growth traits, average daily gain pre test (DGP), average daily gain on test (DGT), and average daily gain through the whole life from birth to the end of test (DGW), were used for population C (Table 1). Birth weights were assumed to be zero in order to estimate DGP and DGW. Phenotypic data were measured in the progeny generation only in populations A and C. In population B, phenotypic values from the parental generation were also used. More details are described in the previous papers [6,14].

**Table 1:** Breed, number of animals and phenotypic data

		Number					
Population	Breed	Sire	Dam	Progeny	Mean (SD)		
					P1		P2
					
A	Large White	10	91	326	8.6 (2.1)		9.6 (2.1)

					P1	P3	L
					
B	Yorkshire/Large White Synthetic	10	146	391	9.1 (1.8)	10.3 (1.9)	9.8 (2.1)

					DGP	DGT	DGW
					
C	Large White	11	138	382	472 (54)	986 (115)	642 (63)

P1, P2 and P3 (mm): back fat thickness at 45, 65 and 80 mm from the dorsal midline on the last rib, respectively; L (mm): loin fat thickness; DGP (g): average daily gain from birth to the start of the test (age of 97 days), birth weight is assumed 0; DGT (g): average daily gain during the test (age from 97 to 144 d); DGW (g): average daily gain from birth to the end of the test (age of 144 d), birth weight is assumed 0

### Markers

The genotyped markers (relative distance from the first marker: cM) were S0001 (0.0), SW35 (11.9), SW839 (15.6), S0107 (17.1), SW841 (23.9) and S0073 (28.4) on chromosome 4 for population A. SW1354 (0.0), SWR1078 (8.9), TNFB (27.5), SW2019 (29.3) and S0102 (39.3 cM) on chromosome 7 were genotyped for populations A and B. S0064 (6.4) and SW1344 (17.0) were additionally genotyped on chromosome 7 for population C. The distances between markers were estimated using the mapping software Crimap [26].

### Model and test statistic

#### Mixed model

Our model includes sex as a fixed effect and polygenic and QTL genotypic effects as random effects. Random effects can be estimated simultaneously using relationship matrices (additive genetic relationship matrix for polygenetic effects and identity-by-descent (IBD) matrix for QTL genotypic effect). We followed a two-step method described in [16] for variance component analysis with IBD matrices constructed by a simple deterministic approach [27]. Variance components were estimated using ASReml [28].

Mixed animal models are as follows:

where the vector **y **represents the phenotypic values, **X **is the design matrix for fixed effect, and **Z **is the design matrix for random effects. The remaining vectors are, **u**: polygenic effect, **w**_1 _and **w**_2_: QTL genotypic effect for the first and second QTL, respectively, **e**: residual, and **β**: fixed effect. Sex was used as a fixed effect for growth traits, and both sex and regression on end weight were used as fixed effects for fat traits. Matrices **A **and **I **are an additive genetic relationship matrix [29] for polygenic effects and a unit matrix, respectively. **Q**_1 _and **Q**_2 _are IBD matrices for variance-covariance of the genotypic effect at QTLs 1 and 2, respectively. Polygenic and residual variances are σ^2^_u _and σ^2^_e_. Genotypic variance at QTLs 1 and 2 are σ_w1_^2 ^and σ_w2_^2^, respectively. Phenotypic variance, σ^2^_p_, is σ^2^_u _+ σ_w1_^2 ^+ σ_w2_^2 ^+ σ^2^_e _for the two-QTL model and σ^2^_u _+ σ_w1_^2 ^+ σ^2^_e _for the one-QTL model.

Heritabilities are h_p_^2 ^(= σ^2^_u_/σ^2^_p_) for the polygenic effect, and h_w1_^2^(= σ^2^_w1_/σ^2^_p_) and h_w2_^2^(= σ^2^_w2_/σ^2^_p_) for genotypic effects at QTL 1 and QTL 2, respectively. When a QTL is significant, QTL genotypic effects (**w**_1 _and **w**_2_) at the peak location are converted into allelic effects using a multiplication with genotypic and allelic IBD matrices. The details of this method were described in a previous paper [13].

### Test for significant QTLs

To estimate the presence of a QTL against the null hypothesis (no QTL) at a test position, the likelihood ratio (LR) test statistic LRT = - 2ln(L_0_/L_1_) was calculated, where L_0 _and L_1 _represent the respective likelihood values under the hypothesis of either the absence (H_0_) or presence (H_1_) of a QTL for the one-QTL model. For the two-QTL model, both the presence of two QTLs against the null hypothesis and the difference between one-QTL and two-QTL models were considered [30]. The hypotheses for comparing the presence of one QTL in the first position and two QTLs in both the first and second positions are as follows:

H_1_: QTL in the first position but no QTL in the second position

H_2_: QTLs in both first and second positions.

When comparing the hypothesis of one QTL in the second position with that of QTLs in both the first and second positions, then H_1_: QTL presence in the second position but no QTL in the first position, was applied and H_2 _was as before.

For the hypothesis tests [31,32], a mixture of χ^2 ^distributions with different degrees of freedom was applied. For the two-QTL model, the hypotheses (H_0_: no QTL, H_2_: two QTLs present) were examined with 1/4 χ_0_^2 ^+1/2χ_1_^2 ^+1/4χ_2_^2 ^distribution, and the hypotheses (H_1_: one QTL, H_2_: two QTLs) were tested with 1/2 χ_1_^2 ^+ 1/2χ_2_^2 ^distribution. For the one-QTL model, the hypotheses (H_0_: no QTL, H_1_: one QTL present) were examined with 1/2 χ_0_^2 ^+ 1/2χ_1_^2 ^distribution.

### Criterion for pleiotropy

When a QTL is found in a similar genomic region for each of two correlated traits, we can consider that either (1) QTLs for the two traits are linked (linkage) or (2) two traits are controlled by the same gene or genes at that location (pleiotropy). There are some tests to find linked loci that affect a single trait [24,25]. However even if there is no proof of linkage, it does not mean that the trait is controlled by a single pleiotropic locus, because we can still argue that the accuracy and power of the test may be insufficient to discriminate between the two situations. Further information provided from denser markers and/or a larger number of generations may reveal the presence of linked loci. Therefore, we do not have a general statistical test to distinguish a pleiotropic locus from linked loci in our analysis scheme. However, we can indicate a theoretical limit of allelic correlation between two traits in a gamete when it is caused by linkage disequilibrium. If allele frequencies do not change across generations, an allelic correlation is determined by a relative size of linkage disequilibrium D [33]. In a large population, linkage disequilibrium of the base population, D_0_, changes to D_t _at generation t with a recombination rate c [19].

Thus, even if there is a perfect correlation (= |1|) between two alleles at linked loci on a gamete at the foundation of a population, it will be reduced by random mating in future generations unless linkage is complete (*i.e*. c = 0.0). For example, population A has been closed for at least 15 generations. In this population, if c is 0.01, (meaning 1 cM between loci), the size of D relative to the base generation is reduced to 0.86 with t (=15) generations and allelic correlation also changes from 1 to 0.86. If the allelic correlation is more than 0.86 in the current generation, the distance between loci must be less than 1 cM or we can assume a single locus (pleiotropy). In this case we choose the value of allelic correlation greater than 0.86 as a cut off with values above this as being indicative of pleiotropy. We recognize that this choice is somewhat arbitrary and that linked loci may be linked at distances less than 1 cM apart. Note, however, that this is a relatively small window compared with the normal mapping accuracy of QTL studies, for example Gardner *et al*. [33] have summarized that the average confidence interval around QTLs is 15.6 cM based on more than 200 mapped QTLs. Populations B and C have been closed for at least 10 and 16 generations, respectively. Thus, we chose as criteria values of the allelic correlation of 0.90 (1 cM linkage distance) and 0.82 (2 cM) in population B and 0.85 (1 cM) and 0.72 (2 cM) in population C. The relatively few parents genotyped only represent a small proportion of the whole breeding populations. Therefore, we assumed that effective population sizes are large enough to apply these criteria.

## Results

### Two QTLs with the two-QTL model (Population A)

**Figure 1:**
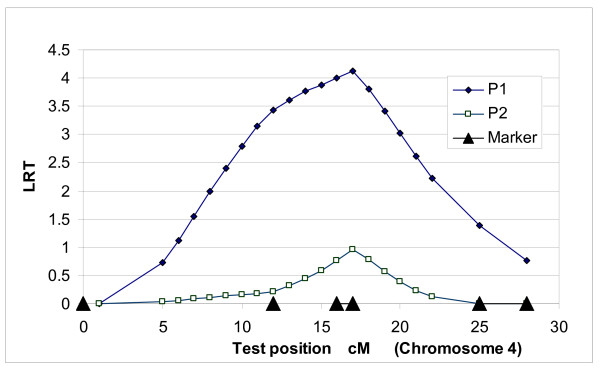
**QTL position for P1 and P2 fat on chromosome 4 using the one-QTL model in population A**.

**Figure 2:**
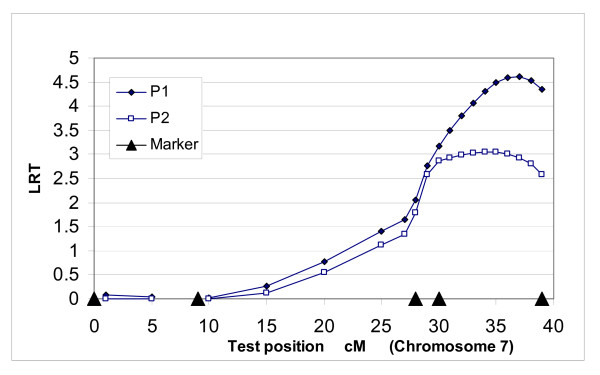
**QTL position for P1 and P2 fat on chromosome 7 using the one-QTL model in population A**.

When the two-QTL model was applied within chromosome 4 and 7, we observed very slight differences in test statistic values between the one-QTL and two-QTL models. Significant differences between the two models were evident when two single loci in the two chromosomes were used. When one-QTL model was applied for P1 on chromosomes 4 and 7, both chromosomes showed QTLs at the 5% significance level (Table 2). The test statistic peaks were at 17 cM on chromosome 4 and 37 cM on chromosome 7 with the one-QTL model (Figures 1 and 2). In the two-QTL model, the peak on chromosome 4 was consistent at 17 cM, but shifted slightly to 34 cM on chromosome 7. The two-QTL model displayed a higher test statistic *i.e*. 10.32, which was significant against both the one-QTL model using chromosomes 4 and 7 (<0.05), and the null hypothesis, no QTL (<0.01) (Table 2). Here, we call the QTLs on chromosomes 4 and 7 'QTL1 and QTL2', respectively. The percentages of residual (one-QTL model: 38% and 41%, two-QTL model: 43%) and QTL genetic variances (QTL1: 14% to 17% and QTL2: 18% to 21%) were slightly altered from the one-QTL to the two-QTL model. However, polygenic variances were reduced from 48% (chromosome 4) and 41% (chromosome 7) to 19% (Table 2).

**Table 2:** One-QTL and two-QTL models for P1 fat in population A

	One QTL		Two QTL
	
	Chromosome 4	Chromosome 7	Chromosome 4 and 7
Position (cM)	17	37	17 and 34^(a)^
LR test statistic	4.1*	4.6*	10.3 ^(b)^*^, (c)^**
Residual (%)	38	41	43
Heritability polygene (%)	48	41	19
QTL1 ^(d) ^(%)	14	-----	17
QTL2 (%)	-----	18	21

* <0.05, ** <0.01; (a) QTL positions are at 17 cM and 34 cM on chromosome 4 and 7, respectively; (b) H_1 _and H_2 _hypothesis; (c) H_0 _and H_2 _hypothesis; (d) QTL1 and QTL 2 on chromosomes 4 and 7, respectively

The one-QTL model for P2 on chromosome 7 was significant (P < 0.05) (Table 3). Test statistic peaks for P2 were lower than those for P1 (Figures 1 and 2). However, the peak for P2 (17 cM) on chromosome 4 was similar to that for P1. The P2 peak on chromosome 7 was at 35 cM, which was very close to the peak at 37 cM for P1. Polygenic heritabilities in the two-QTL model were much lower than those in the one-QTL model (Tables 2 and 3).

**Table 3:** One-QTL and two-QTL models for P2 fat in population A

	One QTL		Two QTL
	
	Chromosome 4	Chromosome 7	Chromosome 4 and 7
Position (cM)	17	35	17 and 32^(a)^
LR test statistic	1.0 ^ns^	3.1*	4.8 ^(b) ns, (c)^*
Residual (%)	32	32	33
Heritability polygene (%)	62	54	44
QTL1 ^(d) ^(%)	6	----	9
QTL2 (%)	----	14	14

* <0.05, ns: non significant; (a) QTL positions are at 17 cM and 32 cM on chromosomes 4 and 7, respectively; (b) H_1 _and H_2 _hypothesis; (c) H_0 _and H_2 _hypothesis; (d) QTL1 and QTL 2 on chromosomes 4 and 7, respectively

Therefore, the estimated total genetic variance, a sum of polygenic and QTL genotypic variances, was similar between the one-QTL and the two-QTL models. For instance, the genetic variance for P2 was 68% (= 62+6) and 68% (= 54+14) in the one-QTL model and 67% (= 44+9+14) in the two-QTL model (Table 3).

### Pleiotropy with the two-QTL model (Population A)

**Figure 3:**
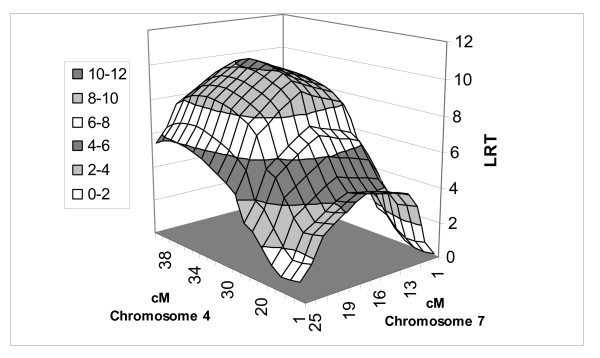
**QTL position for P1 fat using two-QTL model in population A**. Three-dimensional view of QTL on chromosomes 4 and 7.

**Figure 4:**
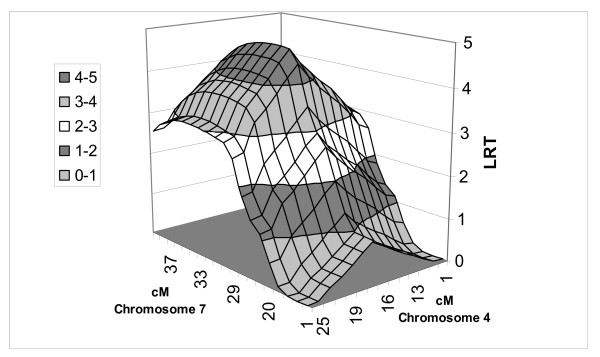
**QTL position for P2 fat using two-QTL model in population A**. Three-dimensional view of QTL on chromosomes 4 and 7.

LR test statistic (Figures 1, 2, 3 and 4) curves showed that figures for P1 and P2 were quite similar on chromosomes 4 and 7. QTLs for both P1 and P2 displayed the same peak at 17 cM on chromosome 4. However, the peaks positions on chromosome 7 were slightly different between models and traits, *i.e*. 37 cM in the one-QTL model and 34 cM in the two-QTL model for P1, and 35 cM in the one-QTL model and 32 cM in the two-QTL model for P2. The differences in test statistic values around these peaks were very small.

The correlation of the allelic effects between P1 and P2 within the gamete from the same parent was highly positive. For instance, the correlation between P1 and P2 was 0.90 within the paternal and 0.94 within the maternal gamete on chromosome 4 (Table 4). These allelic correlations are higher than the criterion of 0.86 calculated earlier for an assumed 1 cM linkage distance. We also examined the allelic correlations between the paternal and maternal gametes for each trait. For instance, the correlation between the paternal allelic effect of P1 and maternal allelic effect of P1 was 0.17 on chromosome 4. These allelic correlations between paternal and maternal gametes were 0.12 for P2 (chromosome 4), 0.13 for P1 and 0.13 for P2 (chromosome 7). They suggest weak assortative mating within a line, which may contribute to the allelic correlation in the same gamete. Therefore, we calculated the partial correlations [34] using the matrix of correlations between all related paternal and maternal allelic effects. The partial correlations between allelic effects on the same gamete allow us to exclude the effect of assortative mating. However, all allelic correlations in the same gamete showed very little variation (maximum change <0.3%).

**Table 4:** Correlations of allelic effects for P1 and P2 fat on chromosome 4 (below diagonal) and chromosome 7 (above diagonal) in Population A

		P1		P2	
	
		P-allele	M-allele	P-allele	M-allele
P1	P-allele	----	0.13	**0.92**^‡^	0.13
	M-allele	0.17	----	0.11	**0.91**^‡^
					
P2	P-allele	**0.90**^‡^	0.13	----	0.13
	M-allele	0.16	**0.94**^‡^	0.12	----

Allelic correlations within a gamete in gothic; Chr 4 and Chr 7: chromosome 4 and chromosome 7; P-allele: allelic effects from paternal gamete; M-allele: allelic effects from maternal gamete; ^‡^: larger than the criterion 0.86 for pleiotropy (less than 1 cM linkage distance)

### Pleiotropy with the one-QTL model (Populations B and C)

**Figure 5:**
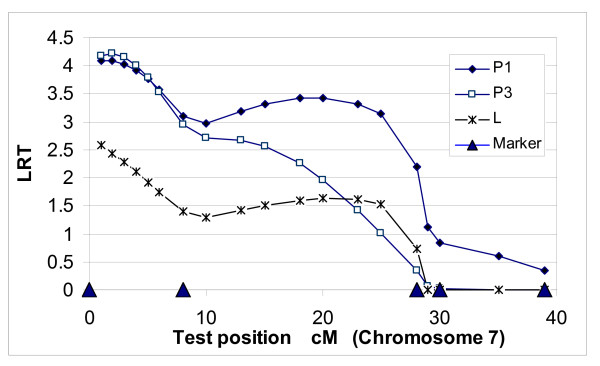
**QTL position for P1, P2 and L fat on chromosome 7 using one-QTL model in population B**.

Three fat traits, P1, P3 and L, from population B, displayed similar peak positions (1 and 2 cM), and two fat traits, P1 and P3, showed significant test statistics (<0.05) on chromosome 7 (Figure 5). The heritabilities for QTLs were 12%, 19% and 10% for P1, P3 and L, respectively (Table 5).

**Table 5:** One-QTL model for P1, P2 and L fat on chromosome 7 in population B

	P1	P3	L
Position (cM)	1	2	1
LR test statistic	4.1 *	4.2 *	2.6
Residual (%)	65	59	71
Heritability polygene (%)	23	22	19
QTL (%)	12	19	10

* <0.05

Allelic effects of the three fat traits within the gamete from the same parent were highly correlated. Two of the allelic correlations between P1 and P3 0.92 and between P1 and L 0.91 within the paternal gamete were higher than a criterion of 0.90 (1 cM linkage distance). Allelic correlations 0.84 within the maternal and 0.86 within the paternal gamete were higher than a criterion of 0.82 (2 cM linkage distance) (Table 6). Phenotypic correlations between three fat traits were also very high (0.81 to 0.89). However, the allelic correlations between paternal and maternal alleles between traits were quite low, *e.g*. 0.09 and 0.16 between P1 and P3 fat. In view of these data (Figure 5) and high allelic and phenotypic correlations, we can conclude that it is likely that the three fat traits are controlled by a single pleiotropic locus.

**Table 6:** Correlations of allelic effects for three fat traits on chromosome 7 in population B

		P1		P3		L	
		P-allele	M-allele	P-allele	M-allele	P-allele	M-allele
P1	P-allele	----	0.14	**0.92**^‡^	0.09	**0.91**^‡^	0.14
	M-allele	----	----	0.16	**0.84**^†^	0.15	**0.81**
							
P3	P-allele			----	0.09	**0.86**^†^	0.14
	M-allele			----	----	0.09	**0.77**
							
L	P-allele					----	0.16
	M-allele					----	----

Allelic correlations within a gamete in gothic (diagonal); P-allele: allelic effects from paternal gamete; M-allele: allelic effects from maternal gamete; ^‡^: larger than the criterion 0.90 for pleiotropy (less than 1 cM linkage distance); ^†^: larger than the criterion 0.82 for pleiotropy (less than 2 cM linkage distance)

**Figure 6:**
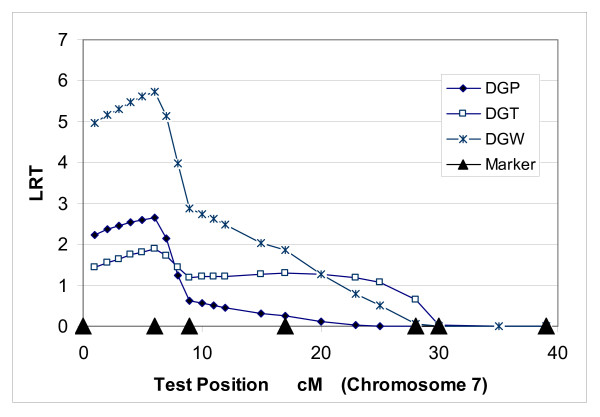
**QTL position for GDP, DGT and DGW growth traits on chromosome 7 using one-QTL model in population C**.

Three measures of growth rate, DGP, DGT and DGW, from population C showed the same peak position, 6 cM, of LR test statistic on chromosome 7 (Table 7 and Figure 6). Heritabilities for QTLs varied from 6% to 12%, and only DGW displayed significant test statistics (<0.01).

**Table 7:** One-QTL model for DGP, DGT and DGW growth traits on chromosome 7 in population C

	DGP	DGT	DGW
Position (cM)	6	6	6
LR test statistic	2.7	1.9	5.7**
Residual (%)	79	45	66
Heritability polygene (%)	12	50	22
QTL (%)	9	6	12

** < 0.01

Allelic correlations within a gamete between DGW and DGP (0.94 and 0.87) are higher than a criterion of 0.85 (1 cM linkage distance). Allelic correlations within a gamete between DGW and DGT (0.82 and 0.71) were also high (Table 8). However, the allelic correlations between DGP and DGT were relatively low (0.64 and 0.41) and they did not reach the criterion 0.72 (2 cM linkage distance). The phenotypic correlation between DGP and DGT (0.41) is also lower than that between DGP and DGW (0.84) and that between DGT and DGW (0.78).

**Table 8:** Correlations of allelic effects for three growth traits on chromosome 7 in population C

		DGP		DGT		DGW	
		P-allele	M-allele	P-allele	M-allele	P-allele	M-allele
DGP	P-allele	----	0.15	**0.64**	0.05	**0.94**^‡^	0.09
	M-allele	----	----	0.11	**0.41**	0.15	**0.87**^‡^
							
DGT	P-allele			----	0.13	**0.82**^†^	0.11
	M-allele			----	----	0.09	**0.71**
							
DGW	P-allele					----	0.11
	M-allele					----	----

Allelic correlations within a gamete in gothic (diagonal); P-allele: allelic effects from paternal gamete; M-allele: allelic effects from maternal gamete; ^‡^: larger than the criterion 0.85 for pleiotropy (less than 1 cM linkage distance); ^†^: larger than the criterion 0.72 for pleiotropy (less than 2 cM linkage distance)

The correlations between paternal and maternal alleles suggest the possibility of weak assortative mating in both populations B and C (Tables 6 and 8). These correlations are very low, *e.g*. 0.14 for P1, 0.09 for P3, 0.16 for L (Table 6). After adjusting for the effect of assortative mating, allelic correlations in the same gamete showed very little variation (maximum change <0.4%) in populations B and C.

## Discussion

The application of a multiple QTL model clarified the genetic properties of our data. The detection of multiple QTLs reduced the polygenic variance, which was previously overestimated in the single QTL model. It is obvious that if we subtract the QTL heritability in one chromosome from the polygenic heritability in the other chromosome in the one-QTL model, the values become quite similar. For instance, if we subtract the heritability of QTL2 (14%) on chromosome 7 from the polygenic heritability of 62% on chromosome 4, it becomes 48% (= 62-14) (Table 3). In a similar manner, the polygenic heritability on chromosome 7 will be 48% (= 54-6). Both reduced polygenic heritability values were 48%, and close to the polygene heritability value (44%) from the two-QTL model. The data suggest that the QTL effects on different chromosomes are included in the polygenic effect when the one-QTL model is applied independently on chromosomes 4 and 7.

Both QTLs, the first and the second, displayed relatively large heritabilities. The QTL heritability varied from 14% to 21% for P1, and from 6% to 14% for P2 in the one-QTL model and the two-QTL model in population A (Table 2 and 3). The sum of QTL heritabilities in the two-QTL model were 38% (= 17 + 21) (Table 2) and 23% (= 9 + 14) (Table 3). These heritabilities are in agreement with previous reports. For instance, de Koning *et al*. [12] have estimated that the QTL heritabilities of fat traits were 8% to 27% across populations and also that heritabilities were high, 12% and 27%, on the different chromosomes for a single fat trait within a population. Evans *et al*. [5] have reported heritabilities of 17% and 8% on different chromosomes in a population. It seems that QTL heritabilities are quite large considering the polygenic heritabilities of the traits concerned [14]. However, note that we can find QTLs only if they have large effects on phenotypic values. QTLs with a small genetic variance cannot be detected in our analysis scheme. For the two-QTL model, positions of interest on the chromosome were searched in two dimensions [3].

When we find QTLs in the same region, we can assume that (1) QTLs for two traits are linked (linkage) or (2) two traits are controlled by genes on the same QTL (pleiotropy). Simulations results have shown that it was not easy to distinguish between these two states [24,35]. Gilbert *et al*. [25] have reported that it is difficult to distinguish two linked QTLs within 25 cM using markers every 10 cM. Even markers every 1 cM were insufficient to distinguish two linked QTLs 5 cM apart [35]. There are some statistical tests for pleiotropy and linkage, which are based on the comparison of different models [24,25,35]. However, if a hypothesis of pleiotropy is chosen because this hypothesis cannot be rejected in favour of the linkage hypothesis (*i.e*. H_0_: Pleiotropy, H_1_: Linked loci) under their experiment conditions (*e.g*. limited number of markers), it might be too easy to conclude on pleiotropy. Gilbert *et al*. [23] have reported a pleiotropic QTL for a fat trait using various models in an F_2 _pig cross population. However, only 10 markers were mapped over 160 cM on chromosome 7. Considering their simulation results [25], which demand at least two markers between linked QTLs, it might be difficult to conclude that they would detect pleiotropy in a real data set.

Closed populations as used in our paper, are less susceptible to show problems of linkage disequilibrium between linked QTLs. However, mixing a population, *e.g*. an F_2 _population from two divergent selection populations, can lead to strong linkage disequilibrium between linked QTLs.

Disequilibrium gives an advantage to F_2 _populations for detecting QTLs because it results in particular relationships between marker and genes at the QTL. On the other hand, disequilibrium between genes on a QTL and another QTL makes their positions and effects more difficult to estimate accurately [24,25,35].

In the present paper, we do not provide a general statistical test to distinguish pleiotropy from linked loci. However, we use a criterion, which is based on the distance of linked QTLs (1 and 2 cM), to investigate pleiotropy using allelic correlation. The choice of the distances (1 and 2 cM) is somewhat arbitrary, however, it is quite a restrictive criterion compared with the normal mapping accuracy of QTL studies. The average confidence interval around QTLs is more than 15 cM in the previous report [33]. In this paper, we have modeled a simple pleiotropic QTL with alleles affecting two traits with the same direction and having an ideal allelic correlation (=1) in a gamete at the foundation of the population. This is a very restrictive condition since allelic values of a trait are completely correlated to the allelic values of the other trait. If we assume a lower allelic correlation (<1) for the founder generation, the criterion to accept pleiotropy would be lower. Our criterion assuming a high allelic correlation in a gamete can be applied to obviously related traits, (*e.g*. a series of fat traits). In practice, however, there are various types of pleiotropic effects on multiple traits. It is not guaranteed that the allelic correlation is highly positive even if two traits are controlled by a single QTL [24,25]. It would be very difficult to detect all types of pleiotropic QTLs in a marker mapping scheme.

## Conclusion

The application of a multiple QTL model in real data set is useful in determining the genetic properties of traits, even if loci are located on different chromosomes. Detection of the second QTL in a model reduced the polygenic heritability and it should improve the accuracy in the estimation of heritabilities for both QTLs. Accurate variance estimation on both polygenes and QTL genes could improve selection schemes for animals. Our results suggest that pleiotropy is not a rare phenomenon among highly related traits (*e.g*. obesity traits).

## Competing interests

The authors declare that they have no competing interests.

## Authors' contributions

YN carried out the analysis and drafted the manuscript. CSH helped to carry out the study and drafting the manuscript. PMV and CSH prepared the data and PW developed the computer program. All authors have read and approved the final manuscript.
